# Bubble Swelling in Ferritic/Martensitic Steels Exposed to Radiation Environment with High Production Rate of Helium

**DOI:** 10.3390/ma14112997

**Published:** 2021-06-01

**Authors:** Stanislav Sojak, Jarmila Degmova, Pavol Noga, Vladimir Krsjak, Vladimir Slugen, Tielong Shen

**Affiliations:** 1 Institute of Nuclear and Physical Engineering, Faculty of Electrical Engineering and Information Technology, Slovak University of Technology in Bratislava, Ilkovičova 3, 81219 Bratislava, Slovakia; jarmila.degmova@stuba.sk (J.D.); vladimir.krsjak@stuba.sk (V.K.); vladimir.slugen@stuba.sk (V.S.); 2 Advanced Technologies Research Institute, Faculty of Materials Science and Technology in Trnava, Slovak University of Technology in Bratislava, Jána Bottu 25, 91724 Trnava, Slovakia; pavol.noga@stuba.sk; 3 Institute of Modern Physics Lanzhou, Chinese Academy of Sciences, Lanzhou 730000, China; shentielong@impcas.ac.cn

**Keywords:** RAFM steels, swelling, helium implantation, helium bubbles, helium embrittlement

## Abstract

Reduced-activativon ferritic/martensitic (RAFM) steels are prospective structural materials for fission/fusion nuclear applications because their radiation and swelling resistance outperforms their austenitic counterparts. In radiation environments with a high production rate of helium, such as fusion or spallation applications, these materials suffer from non-negligible swelling due to the inhibited recombination between vacancy and interstitial-type defects. In this work, swelling in helium-implanted Eurofer 97 steel is investigated with a focus on helium production rates in a wide range of helium/dpa ratios. The results show virtually no swelling incubation period preceding a steady-state swelling of about 2 × 10^−4^%/He-appm/dpa. A saturation of swelling above 5000 He-appm/dpa was observed and attributed to helium bubbles becoming the dominant sinks for new vacancies and helium atoms. Despite a relatively low irradiation temperature (65 ± 5 °C) and a rather high concentration of helium, transmission electron microscope (TEM) results confirmed a microstructure typical of ferritic/martensitic steels exposed to radiation environments with high production rates of helium.

## 1. Introduction

Volumetric void, or bubble swelling, is one of the major degradation mechanisms of materials exposed to harsh radiation conditions. It is a key factor limiting the safe operational lifetime of nuclear power plants (NPP) and other nuclear installations. Currently, austenitic steels are used as structural materials at NPPs but their low swelling resistance to neutron irradiations limits their application in environments with high temperatures and dpa rates, which is expected for future nuclear facilities. Similar behaviour can also be observed at irradiation by charged particles [1].

Among structural materials, reduced-activation ferritic/martensitic (RAFM) steels are known for their high resistance to void swelling. Due to their additional low thermal expansion and high thermal conductivity, they are considered as candidate materials for applications in nuclear fusion reactors. RAFM steel Eurofer 97 was chosen as the main blanket structural material for the experimental fusion reactor DEMO.

Substantial research activities are currently underway to develop advanced steels with higher radiation resistance. Physical and mechanical properties of new fabricated model alloys are being tested in harsh radiation environments, such as in spallation neutron targets [2]. The main goal of these research studies is to increase the radiation damage resistance of nuclear structural materials, leading to a long operational lifetime and reliability of fusion reactors [3].

Even though the void swelling resistance of ferritic/martensitic (F/M) steels is fairly adequate, their resistance to bubble swelling after exposure to radiation with high production rates of helium (fusion or spallation) is still questionable. A study on the bubble swelling of 9% Cr and 12% Cr RAFM steel after irradiation to 400 dpa by Fe^++^ at 460 °C, was published by Wang et al. [4]. Results reported 20–300 times lower swelling for 12% Cr steel after irradiation.

This decrease was dependent on the presence of sinks (grain/lath boundaries), oxides, and radiation-induced voids which influence the swelling rate [5,6]. The harsh environment of deuterium–tritium (DT) fusion [3] will likely lead to a very different swelling response compared to that of a fission reactor. Therefore, in-depth microstructural studies are needed to address the swelling phenomenon to improve the radiation resistance of materials to be used in future fusion reactors.

The objective of the study is twofold: to assess the potential of implanting a single-beam ion (helium) to simulate spallation and spallation-relevant radiation environments and to provide a better understanding of the sole effect of the helium production rate on volumetric bubble swelling in F/M steels. To achieve these objectives, we used a low temperature (65 ± 5 °C) helium injection and cross-sectional transmission electron microscope (TEM) analysis. The results are discussed with respect to the helium-to-dpa (*c_He_*/dpa) ratio to investigate the performance of F/M steels in a wide range of harsh radiation environments.

## 2. Experimental

In the present experiments, the RAFM steel “Eurofer 97” (Fe-8.9Cr-1.1W-0.47Mn-0.2V-0.14Ta-0.11C), was investigated. A 10 × 10 × 0.5 mm sample was prepared by electrical-discharge cutting and mechanical polishing. To eliminate any residual stresses induced by the polishing, the surface was given an electrochemical polishing in a perchloric acid solution. The sample was subsequently irradiated at a temperature of 65 ± 5 °C with 500 keV He+ ions with a fluence of 10^18^ ions/cm^2^ using the 500 kV open-air implanter at ATRI MTF STU [7]. The number of displacements per atom (dpa), calculated according to the NRT model [8] ranging from 1.6 to 39.4 for the near-surface region (50 nm) and peak region (1050 nm), respectively. The helium concentration (*c_He_*) determined in the same way was 370 and 50 atomic parts per million (appm) at.%, respectively. The range of *c_He_*/dpa ratios studied in this work was from 460 to 15,000 He-appm/dpa. For a comparison, typical helium production rates in FeCr steel are ~10 He-appm/dpa under fusion reactor conditions and up to 100 appm/dpa in spallation neutron targets [9].

The cross-sectional TEM samples of the implanted steel were prepared using the focused ion beam (FIB) lift-out technique using a FEI Helios Nanolab system (Nanolab Technologies, Milpitas, CA, USA), with Ga acceleration voltage between 1 kV to 30 kV. In the next step, a cleaning process to remove the FIB damaged layer was performed by low energy (Ga) ion milling. Microstructural characterization was performed using FEI Tecnai F20 TEM (FEI Company—Thermo Fisher Scientific, Hillsboro, OR, USA), operated at an accelerating voltage of 200 kV with a field emission gun.

**Figure 1:**
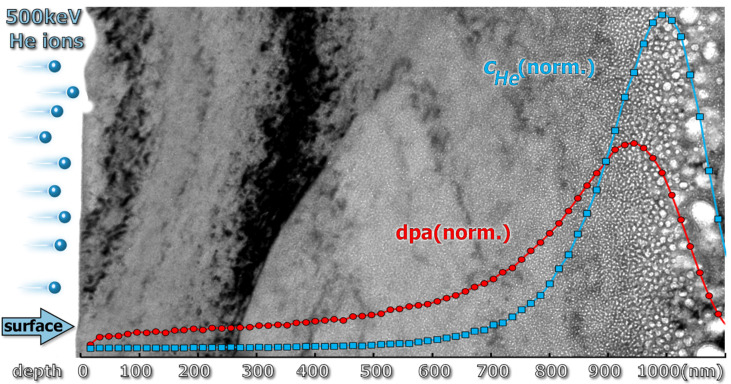
Theoretical simulation of the dpa and *c_He_* profiles with a cross-sectional transmission electron microscope (TEM) micrograph of the implanted Eurofer 97 steel in the background.

The dpa profile and injected helium concentration profiles, normalized to the area under the curves, are shown in Figure 1 with a cross-sectional TEM micrograph of the implanted steel in the background.

## 3. Results

**Figure 2:**
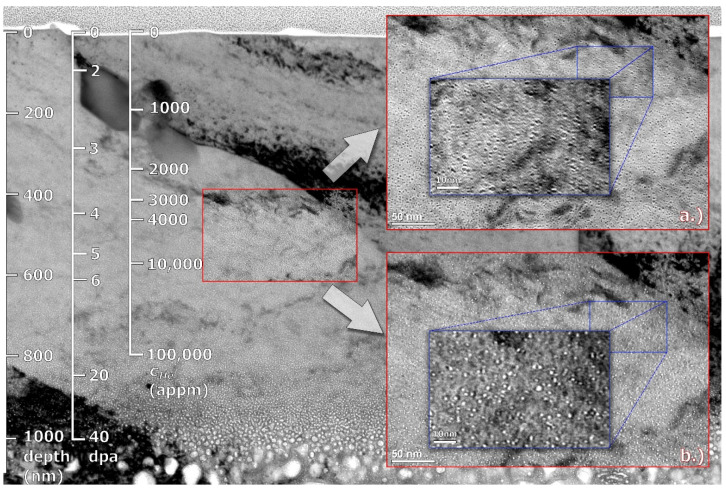
Cross-sectional bright-field TEM micrograph of the implanted steel with magnified dark-field (**a**) and bright-field (**b**) insets representing the range of 3000–10,000 He-appm.

Previously published TEM studies of Eurofer 97 steel [10,11] identified martensite laths of a microns to about 20 µm, with present carbide precipitates along the grain boundaries (M_23_C_6_) and inside the martensite grains (MX). Our TEM observation in Figure 2 shows a bright field cross-sectional micrograph of the He-implanted Eurofer 97 steel. As can be seen in the figure, the radiation-induced defects start to be resolvable at a depth of >300 nm, i.e., at ~3 dpa, or ~2000 He-appm, respectively. Two magnified insets, dark field (a) and bright field (b), shown in Figure 2, suggest a relatively uniform microstructure across the mapped area. This area represents very different implantation conditions for helium concentration (*c_He_*) and displacement damage (dpa). This implicitly points to the role of the irradiation temperature, a dominant factor in swelling, caused by neutron (produced either in fission or spallation reactions) irradiation in structural steels. Since the temperature is basically constant across the implantation profile, a 200–300% increase in *c_He_* and dpa does not lead to a very significant change in microstructure because the F/M steels do not swell at such low displacement damage (~3 dpa) even at high temperatures (>350 °C) [12]. Therefore, the main driving force for volumetric growth is associated with interstitial helium. Another perspective can be obtained from using the ratio of *c_He_* and dpa parameters, which change only slightly in the given area. This suggests that the (*c_He_*/dpa) parameter is a better indicator (predictor) of volumetric bubble swelling than either displacement damage (dpa) or helium concentration (*c_He_*).

**Figure 3:**
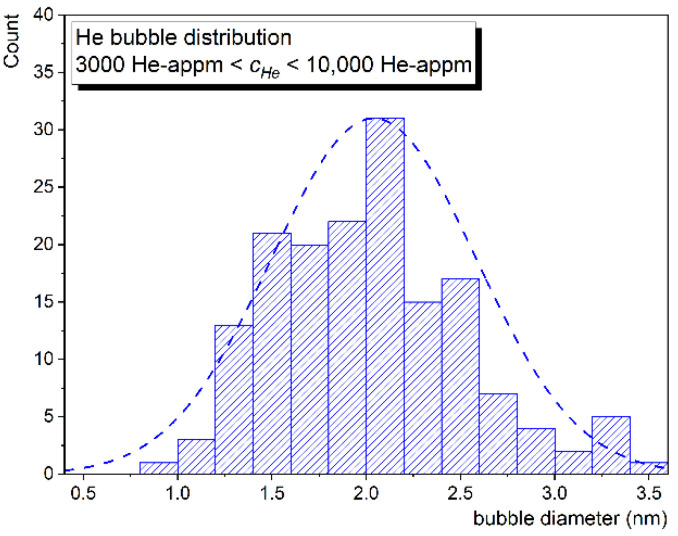
Size distributions of helium bubbles corresponding to *c_He_* range 3000–10,000 appm.

The distribution of bubble size corresponding to the region with He concentration 3000–10,000 appm is shown in Figure 3. With average size of ~2 nm the microstructure can be compared to similar F/M steels (F82H) irradiated in a spallation neutron target to ~20 dpa at an irradiation temperature ~300 °C and with a transmutation helium concentration of ~1800 appm [13]. It is clear from the cross-sectional TEM image that the size of bubbles grew significantly (>5 nm) towards the dpa and *c_He_* peak.

**Figure 4:**
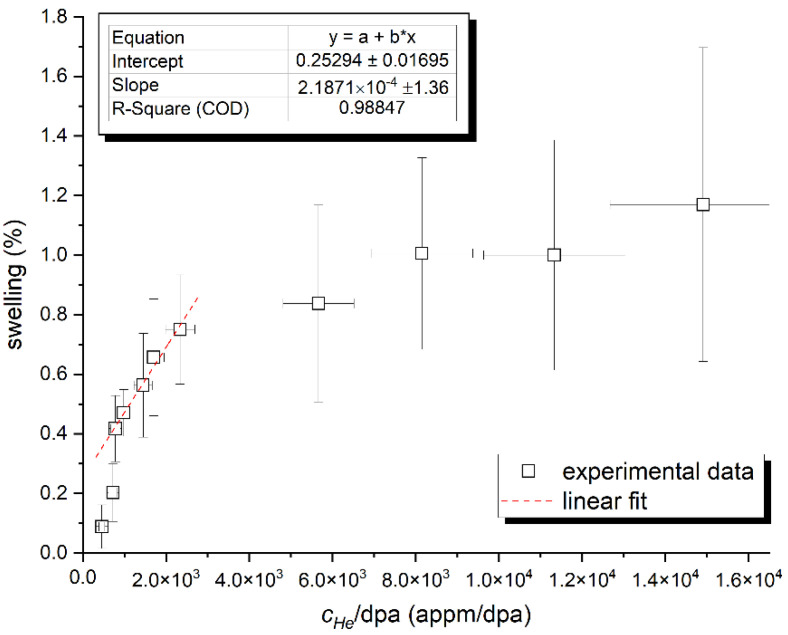
Volumetric bubble swelling in Eurofer 97 steel as a function of helium concentration relative to the number of displacements per atom (*c_He_*/dpa).

In total, 11 areas of at least 50 × 50 nm were analyzed at a depth ranging from 350 to 900 nm, representing the range of 460–15,000 He-appm/dpa. Since the main parameter affecting low-temperature bubble swelling in helium-implanted ferritic steels is the concentration of helium *c_He_*, rather than the dpa, it is reasonable to evaluate the swelling rate with respect to the *c_He_* or *c_He_*/dpa ratio, respectively. Figure 4 shows the volumetric bubble swelling associated with the helium production rate. Since only a weak dependence of the swelling rate on the dpa was expected, it was evaluated only as an average value, i.e., 0.08 ± 0.03%/dpa. This was, in the first approach, surprisingly low with respect to a commonly accepted steady state swelling rate of 0.2%/dpa for F/M steels irradiated under fast reactor conditions [14]. On the other hand, the experimental swelling data in Figure 4 show virtually no transient regime (incubation period) prior to the onset of steady-state swelling, which can be clearly seen between 750 and 3000 He-appm/dpa. The incubation period is subsequently represented by the initial two data points in the figure. The experimental swelling data were saturated after approximately 3000 He-appm/dpa. This seemed to represent the irradiation conditions where newly formed bubbles were becoming dominant sinks for radiation-induced vacancies and helium. Such a regime would, in material engineering, correspond to massive damage, likely compromising the structural integrity of the given component. Nevertheless, considering the general trend in the plotted data, volumetric bubble swelling in the studied steel could be described as a nonlinear saturation using either logarithmic or a 1-exp function. A more comprehensive assessment of helium bubble evolution in a wide range of He concentrations, that includes complementary positron annihilation experiments and different types of Fe–Cr alloys, is being carried out.

**Figure 5:**
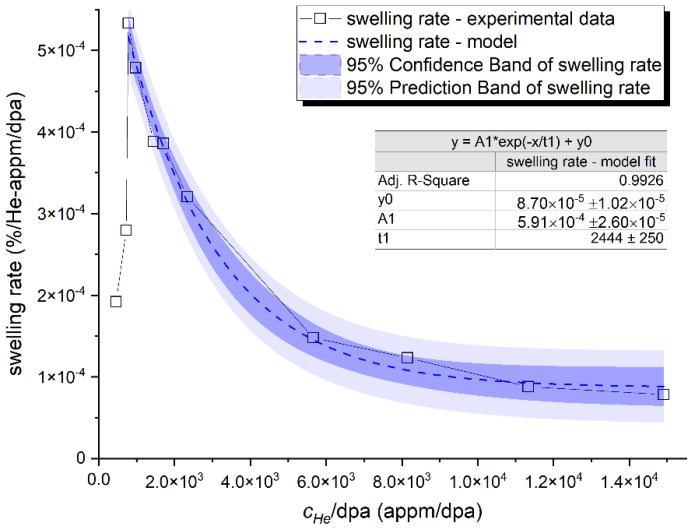
Swelling rate relative to the *c_He_*/dpa ratio with a model fit to the data representing the volumetric bubble swelling in F/M steels exposed to radiation environments with high production rates of helium.

The increasing dominance of helium bubbles as sinks for radiation-induced vacancies and helium atoms is illustrated by a decrease swelling rate along the increase of the *c_He_*/dpa ratio in Figure 5. The incubation period can be very well resolved in the plot (first two data points). Therefore, to describe analytically the role of helium production rate in the volumetric bubble swelling, we used the data plotted in Figure 5.

The following behavior, steady-state swelling and the saturation of the swelling, can be very well approximated by a swelling rate exponential decay formula $A*exp\left(-x/t_{1}\right)+y_{0}$, where A, t_1_ and y_0_ are approximately equal to 5.9 × 10^−4^%/He-appm–/dpa, 2400 He-appm/dpa and 9 × 10^−5^%/He-appm/dpa, respectively.

## 4. Conclusions

The present study provided a new perspective on the behaviour of reduced-activation ferittic/martensitic (RAFM) steels exposed to radiation environments having a high production rate of helium. A wide range of radiation conditions in helium production rates was investigated using a single-beam helium implantation and cross-sectional TEM analysis. Despite relatively low irradiation temperature (65 ± 5 °C) and a rather high helium concentration, the TEM showed a microstructure typical for RAFM steels exposed to harsh radiation environments. This suggested that low-temperature helium implantation can be a valuable tool in an experimental simulation of challenging radiation conditions, such as the environment of fusion reactors or spallation neutron sources. Our study suggested that a suitable depth-sensitive technique can provide unique data on helium-implanted structural materials to improve understanding of the role that the helium production rate plays on the evolution of the microstructure.

The results show virtually no swelling incubation period preceding a steady-state swelling of about 2×10^−4^%/He-appm/dpa. A saturation of swelling above 5000 He-appm/dpa was observed and attributed to helium bubbles, which become dominant sinks for new vacancies and helium atoms. The results indicated that a high helium concentration introduced in near-room temperature implantations had a similar effect on the high irradiation temperature. The saturation of the volumetric swelling of helium-containing irradiated material, therefore, can be interpreted solely neither by displacement damage nor by the irradiation temperature, and all available information on helium concentration must be carefully considered.

## Data Availability

All data used to reach the conclusions are presented in the paper. TEM micrographs are available upon request from the corresponding author.
